# Comparative performance of four malaria rapid diagnostic tests, Vikia Malaria *Pf*/Pan, Meriline-Meriscreen *Pf/Pv*/Pan, Right Sign Malaria *Pf*/Pan, and Right Sign Malaria *Pf*, among febrile patients in Gabon

**DOI:** 10.1590/0037-8682-0274-2019

**Published:** 2020-06-22

**Authors:** Bridy Chesly Moutombi Ditombi, Julienne Isabelle Minko, Roméo Philippe Tsamba, Jacques Marie Ndong Ngomo, Tatiana Nymane, Fanny Bertrande Batchy Ognagosso, Noé Patrick M’bondoukwe, Denise Patricia Mawili-Mboumba, Marielle Karine Bouyou Akotet

**Affiliations:** 1 Université des Sciences de la Santé, Faculty of Medicine, Department of Parasitology-Mycology, BP 4009 Libreville, Gabon.; 2 Regional Hospital of Melen, Clinical and Operational Research Unit, Estuaire Gabon.; 3 Université des Sciences de la Santé, Faculty of Medicine, Department of Paediatrics, BP 4009 Libreville, Gabon.

**Keywords:** RDT, Performance, Malaria, Gabon

## Abstract

**INTRODUCTION:**

Rapid diagnostic tests (RDTs) are selected based on their performances. Here, we compared the diagnostic performance of different malaria RDTs.

**METHODS:**

Febrile patients were tested for malaria using Vikia Malaria *Pf*/Pan, Meriline-Meriscreen *Pf/Pv*/Pan, Right Sign Malaria *Pf*/Pan, and Right Sign Malaria *Pf* RDTs at Melen Regional Hospital in Gabon.

**RESULTS:**

In total, 120 of 274 tested children (43.8%) had malaria. The sensitivity was > 95% for all RDTs, while the specificity was > 85% for two tests. One test generated invalid tests (8%).

**CONCLUSIONS:**

Based on their performances, all tests except one may be recommended for malaria diagnosis.

The World Health Organization (WHO) recommends the use of microscopic examination or rapid diagnostic tests (RDTs) for the biological diagnosis of malaria in febrile patients before the prescription of antimalarial drug treatment^1^. Malaria RDTs are now implemented in several endemic countries, particularly in remote areas where trained staff and microscopes are unavailable to carry out accurate malaria diagnoses. In the last decade, the number of different RDTs has considerably increased^2^. These have been provided for free in many countries^3^. As a result, the percentage of febrile patients benefitting from such tests has increased from 40% in 2010 to 76% in 2015. In line with the WHO recommendations, the Ministry of Health in Gabon has adopted the use of RDTs for malaria diagnosis in public health centers. However, specific RDTs are not yet recommended by the Malaria National Control Program. While different RDTs are currently available in the country, the performances of Meriline-Meriscreen or RDTs with modified trade names such as Right Sign *Pf/*Pan and Right Sign *Pf* have never been evaluated. In the present study, we compared the performances of four different RDTs for malaria diagnosis, namely, VIKIA Malaria *Pf*/Pan, Meriline-Meriscreen *Pf/Pv*/Pan, Right Sign Malaria *Pf*/Pan, and Right Sign Malaria *Pf* kits. The comparison was carried out between these tests and previous data. Microscopy was used as the gold standard method.

This cross-sectional study was performed between April and June 2016 at the Clinical and Operational Research Unit located at the Melen Regional Hospital (RHM), a sentinel site for malaria survey in Gabon. The patients examined in this study had a temperature - > 37.5°C or a history of fever 48 h prior to their visit. The following data were collected after obtaining the patients’ consent to participate in the study: body temperature, fever status, age, sex, and any intake of antimalarial drug before consultation.

Blood (1 mL) samples were collected from each patient in an ethylenediaminetetraacetic acid (EDTA)-coated tube and subjected to malaria diagnosis using RDTs and microscopy by an experienced staff. The Lambaréné’s method was employed for microscopic examination, as previously described by Planche et al^4^.

Three RDTs were in a cassette format. We first tested the VIKIA® Malaria *Pf/*Pan (BioMerieux SA, France) kit that detects the histidine-rich protein-2 (HRP-2) antigen specific to *Plasmodium falciparum* and aldolase, an enzyme common to all *Plasmodium* species. Its sensitivity is known to range from 89.1%^5^ to 96.3%^6^. 

We then tested the Meriline-Meriscreen Malaria *Pf/Pv*/Pan (Meril Diagnostic, Gujarat, India) kit. In this kit, the ‘Pan’ line is coated with an anti-*Plasmodium* lactate dehydrogenase (pLDH) antibody specific topLDH common to all *Plasmodium* species, the ‘*Pv*’ test line is coated with an anti-pLDH antibody specific for *P. vivax*, while the ‘*Pf*’ test line is coated with an anti-HRP2 antibody. This RDT exhibited a sensitivity of 100%, as reported by the manufacturer.

The Right Sign Malaria *Pf*/Pan test (Biotest, Hangzhou Biotest Biotech Co, China) allows the detection of HRP2 and aldolase. This test, when used with clinical samples, has sensitivity values between 98.0% and 99.9%^7^. 

According to the manufacturer instructions, 5 µL of whole blood samples were dispensed in wells, and three to five drops of the buffer from VIKIA Malaria *Pf*/Pan, Meriline-Meriscreen *Pf/Pv*/Pan, Right Sign Malaria *Pf*/Pan, and Right Sign Malaria *Pf* kits were added into neighboring wells. Results were obtained after 10 to 25 min.

The interpretation of these three tests was similar and carried out according to the manufacturer’s instructions. Negative, *P. falciparum-*positive, *P. vivax, P. ovale*, *or P. malariae*-positive, and/or *P. falciparum* and *Pan*-positive samples were determined according to the type and number of colored bands. Results were not validated when no control line was displayed.

The Right Sign Malaria *Pf* test (Biotest, Hangzhou Biotest Biotech Co, China) captures the HRP2 antigen on the strip. Its sensitivity is >99.0%. Whole blood (10 µL) was added into a tube and mixed with three drops of buffer. The test strip was vertically inserted into the tube and the results were obtained after 10 min. Appearance of two colored bands (control line ‘C’ and test line ‘T’) indicated *P. falciparum*-positive result, while a single colored line on the control line ‘C’ corresponded to negative results. Results were invalid in the absence of any color on control line.

RDTs were stored between 19°C and 25°C as per manufacturers’ instructions (5°C-30°C). The integrity of the desiccant was controlled in each box containing RDTs before use. The tests were performed by trained members and experienced laboratory technicians.

Experienced technicians read blood smears and in case of discordance, slides were judged by a third reader. The two closest parasitemia were considered to determine the median parasite density. 

The study was performed at one of the five sentinel sites for malaria survey of the Gabonese Ministry of Health. The Department of Parasitology Mycology served as the reference laboratory for the Malaria National Control Program and has the approval of the health authorities to perform free malaria diagnoses for febrile patients to monitor malaria morbidity and evaluate RDTs at all sentinel sites. After information and appropriate explanations, the parents or legal guardians of all children willing to participate in the study signed an agreement before sampling. 

Data were recorded on a case report form (CRF) and entered in an Excel sheet. Statistical analysis was performed using StatView 5.0 (SAS Institute, Cary, NC, USA). Microscopy was considered as the gold standard method. RDT results were categorized as negative, *P. falciparum* mono-infection, non-*P. falciparum* species infection, and mixed infection (infection with *P. falciparum* and non-*P. falciparum* malaria parasites). Sensitivity (Se), specificity, negative predictive value, positive predictive value, false positivity, and false negativity were assessed and compared. A p-value less than 0.05 was considered significant.

Overall, 274 patients were selected during the study period, among which 50.5% (n = 138) were male. The median age was 5 [1.5-9] years; 49.2% (n = 135) of the participants were less than 5 years old. Among all patients, 77.0% (n = 211) had fever on the day of consultation and the median temperature was 38.5°C (38-39.2°C). Less than one-third of all patients (21.6%; n = 59/274) consumed an antimalarial drug prior to their visit. 

Based on microscopy analysis, almost half of patients (43.8%;n = 120/274) had a positive blood smear (PBS). *P. falciparum* was the only parasite species identified. In the infected patients, the median parasite density was 10,500 (2,083-36,050) P/µL. Parasitemia levels ranged from 35 to 420,000 P/µL. The parasite density in 6 (5%) and 10 (8.3%) patients was less than 500 P/µL and between 500 and 1,000 P/µL, respectively, while 104 (86.7%) patients had more than 1,000 P/µL.

Malaria infection was diagnosed in 51.4% (n = 141/274) of thepatients using VIKIA Malaria *Pf/*Pan kit, 51.8% ofcases (n = 142/274) using Right Sign Malaria *Pf/*Pan kit, and 51.1% ofpatients (n = 140/274) using Right Sign Malaria *Pf* test. The Meriline-Meriscreen *Pf*/*Pv*/Pan test detected 46.0% (n = 126/274) of infected individuals. Invalid results were observed with the Meriline-Meriscreen test (Table 1). 

**TABLE 1:** Results and performances of RDTs.

	**Vikia Malaria *Pf*/Pan**	**Meriline-Meriscreen *Pf/Pv*/Pan**	**Right Sign Malaria *Pf*/Pan**	**Right Sign Malaria *Pf***
Positive n (%)	141 (51.5)	126 (46.0)	142 (51.8)	134 (48.9)
Negative n (%)	133 (48.5)	125 (45.6)	132 (48.2)	140 (51.1)
Invalid n (%)	0 (0.0)	23 (8.4)	0 (0.0)	0 (0.0)
**Species identification**				
*Pf* n (%)	20 (14.2)	0 (0.0)	36 (25.4)	140 (100.0)
*Pf*/Pan n (%)	121 (85.8)	125 (99.2)	105 (73.9)	0 (0.0)
Pan n (%)	0 (0.0)	1 (0.8)	1 (0.7)	0 (0.0)
Sensitivity (%)	97.6	97.2	95.8	97.5
[IC95%]	[94.5-98.8]	[95.2-99.1]	[93.3-98.2]	[95.6-99.3]
Specificity (%)	83.0	86.0	82.5	85.1
[IC95%]	[78.5-87.4]	[81.9-90.1]	[78.8-86.9]	[80.8-89.3]
PPV (%)	82.3	84.1	81.0	83.6
NPV (%)	97.0	97.6	96.5	97.8
LR positive	5.64	5.97	5.27	6.46
LR negative	0.03	0.03	0.06	0.03

More than 95% of PBS were confirmed positive using the RDTs tested. The proportion of false-positive results varied between 14.1% and 17.5% depending on the RDTs. False-negative results were observed in less than 5% of febrile cases.

The sensitivity of RDTs was above 95%, with a value of 97% reported for Right Sign Malaria *Pf* and Meriline-Meriscreen *Pf*/*Pv*/Pan tests. The specificity was above 80% (Table 1). Negative predictive values determined for each RDT ranged from 96.2% to 97.8% (Table 1). The proportion of false-positive cases was higher among patients who took antimalarial drugs prior to consultation than in those who did not take antimalarials. This proportion ranged from 23.7% with Right Sign Malaria *Pf* kit to 28.9% with Right Sign Malaria *Pf*/Pan kit but never exceeded 14% in patients without self-medication (Table 2). 

**TABLE 2:** Relationship between antimalarial drug intake (self-medication) and false-positive (FP) frequency.

		ACT* (+)		ACT* (−)	
		NBS* (n = 38)	FP (%)	NBS (n = 115)	FP (%)
Vikia Malaria *Pf*/Pan	(+)	10	26.3	15	13.0
Meriline-Meriscreen *Pf/Pv*/Pan	(+)	9	23.7	11	10.5
Right Sign Malaria *Pf*/Pan	(+)	11	28.9	16	13.9
Right Sign Malaria *Pf*	(+)	9	23.7	14	12.2

ACT*(+): group of patients on self-medication with artemisinin-based combination therapy. ACT* (−): group of patients without self-medication. NBS*: Negative blood smear.

The sensitivity of VIKIA Malaria *Pf*/Pan and both Right Sign Malaria *Pf* and *Pf*/Pan RDTs ranged from 93.5% to 100% when parasite density was above 1,000 P/µL. Two tests, Vikia and Right Sign *Pf*, detected all infected samples when the parasite density was above 5,000 P/µL (100%). Samples (n = 3) with parasitemia below 200 P/µL were positive with all RDTs. Two samples with a parasite density of 2,800 and 4,900 P/µL were negative with all RDTs (Table 3).

**TABLE 3:** RDT sensitivity according to parasitemia.

RDT sensitivity				
Parasite density	Vikia Malaria	Meriline-Meriscreen (*)	Right Sign Malaria	Right Sign
(in p/μL)	***Pf*/Pan, %**	***Pf/Pv*/Pan, %**	***Pf*/Pan, %**	**Malaria *Pf*, %**
1-500 (n = 6)	83.3	100.0	83.3	83.3
501-1000 (n = 10)	90.0	87.5	90.0	100.0
1001-5000 (n = 31)	93.5	87.1	93.5	93.5
5001-50000 (n = 48)	100.0	93.7	100.0	100.0
> 50000 (n = 25)	100.0	100.0	96.0	100.0

(*) Meriline-Meriscreen *Pf/Pv*/Pan displayed invalid results according to the parasites density, the percentage varied between 6.2% and 33.3%.

In the present study, we compared four malaria RDTs with light microscopy at a sentinel site to evaluate their performances. The sensitivity was above 95% and similar for all RDTs tested. These results are comparable to the previously obtained data in Gabon^8^ ^,^ ^9^. The sensitivity of Right Sign Malaria *Pf* (97.5%) and Meriline-Meriscreen *Pf*/*Pv*/Pan (97.2%) tests tended to be slightly higher than that of VIKIA Malaria *Pf*/Pan (96.7%) and Right Sign Malaria *Pf*/Pan (95.8%) tests. In other malaria endemic countries, RDTs targeting same antigens had lower sensitivity values. For instance, the sensitivity values for the detection of HRP2 and pLDH were 85.7% for Paracheck TM-*Pf*, 88.2% for SD Bioline Ag-*Pf*/Pan, and 90.2% for SD 05FK60^10^, while those for the detection of HRP2 alone were 97% for SD Bioline, 92% for First response malaria, 91% for Parachek, and 85.4% for SD Bioline Ag-*Pf* ^11^. Acceptable sensitivity is imperative to ensure the accurate detection of malaria cases to facilitate the initiation of an appropriate treatment regimen. 

The different RDTs tested herein had relatively low specificity (83.3% for all RDTs) in comparison to the RDTs detecting HRP2 and pLDH in Ethiopia (98.6%)^12^. The loss in specificity could be attributed to the detection of HRP2 circulating antigens, which may persist in the blood for several weeks after malaria treatment. In the present study, almost a quarter of all patients used anti-malarial drugs prior to consultation; one-third of patients were detected positive by all RDTs. Moreover, RDTs were twice more positive among patients who used self-medication than those who did not.

The proportion of false-positive cases was also similar between all kits and ranged from 14% to 17%, although the Right Sign Malaria *Pf*/Pan kit had the highest frequency of false-positive cases (17.5%). This observation may be related to the release of parasite antigens or undetectable asexual parasite density or gametocytemia by all tests. This proportion of false positive cases is high, given that this rate should be less than 10% for all the RDTs selected^13^. 

Right Sign Malaria *Pf*/Pan and Right Sign Malaria *Pf* tests have already been assessed for malaria diagnosis in Gabon under the trade name Acon®^9^ RDT. However, the performances of Right Sign *Pf*/Pan^9^ and Right Sign *Pf* tests were similar to those reported 4 years ago. 

*P. falciparum* was the only species detected by microscopy and RDTs; one patient was diagnosed with a non-*P. falciparum* infection using two RDTs. The WHO/RDT/FIND/CDC recommends a detection score of *P. falciparum* in at least 75% samples with a parasite density of 200 parasites/µL^13^. Here, all RDTs had a sensitivity of 100% when the parasite density ranged from 1 to 200 P/µL. Factors such as low parasitemia that affect RDT sensitivity and specificity pose challenges for malaria diagnosis. 

Meriline-Meriscreen is the only RDT that provided invalid results at a rate of 8.4%, raising concerns related to its storage and transportation. This rate should be less than 5%^13^ based on the selection criteria of WHO for RDT purchase.

Our study has some limitations. Although microscopy remains the gold standard method, molecular techniques for DNA amplification exhibiting high sensitivity values for low parasitemia detection could have provided better estimates of the frequency of false-positive results. However, the procedure of blood smear examination and good quality control of slide reading may have reduced the risk of misdiagnosis.

In conclusion, these RDTs exhibited very good sensitivity (above 95%) despite low specificity (lower than 90%) for the detection of *P. falciparum* or mixed infections. The negative predictive value was above 97% for all tests. The use of these tests will undeniably help in the diagnosis of malaria in situations where microscope is unavailable, with an exception of Meriline-Meriscreen *Pf*/*Pv*/Pan test. This study also highlights the importance of regular RDT surveys before their deployment to identify the possible changes in their on-field accuracy. 
